# Transcriptome Profiling of Human Follicle Dermal Papilla Cells in response to Porphyra-334 Treatment by RNA-Seq

**DOI:** 10.1155/2021/6637513

**Published:** 2021-01-13

**Authors:** Su Yeon Kim, Won Kyong Cho, Hye-In Kim, Seung Hye Paek, Sung Joo Jang, Yeonhwa Jo, Hoseong Choi, Jeong Hun Lee, Sang Hyun Moh

**Affiliations:** ^1^Department of Bio Cosmetics, Sungkyunkwan University, Suwon 16419, Republic of Korea; ^2^Anti-aging Research Institute of BIO-FD&C Co., Ltd., Incheon 21990, Republic of Korea; ^3^Research Institute of Agriculture and Life Sciences, College of Agriculture and Life Sciences, Seoul National University, Seoul 08826, Republic of Korea

## Abstract

Porphyra-334 is a kind of mycosporine-like amino acid absorbing ultraviolet-A. Here, we characterized porphyra-334 as a potential antiaging agent. An *in vitro* assay revealed that porphyra-334 dramatically promoted collagen synthesis in fibroblast cells. The effect of porphyra-334 on cell proliferation was dependent on the cell type, and the increase of cell viability by porphyra-334 was the highest in keratinocyte cells among the three tested cell types. An *in vivo* clinical test with 22 participants demonstrated the possible role of porphyra-334 in the improvement of periorbital wrinkles. RNA-sequencing using human follicle dermal papilla (HFDP) cells upon porphyra-334 treatment identified the upregulation of metallothionein- (MT-) associated genes, confirming the antioxidant role of porphyra-334 with MT. Moreover, the expression of genes involved in nuclear chromosome segregation and the encoding of components of kinetochores was upregulated by porphyra-334 treatment. Furthermore, we found that several genes associated with the hair follicle cycle, the hair follicle structure, the epidermal structure, and stem cells were upregulated by porphyra-334 treatment, suggesting the potential role of porphyra-334 in hair follicle growth and maintenance. In summary, we provided several new pieces of evidence of porphyra-334 as a potential antiaging cosmetic agent and elucidated the expression network in HFDP cells upon porphyra-334.

## 1. Introduction

Mycosporine-like amino acids (MAAs) are small secondary metabolites that were first identified from fungi showing ultraviolet- (UV-) induced sporulation [1]. MAAs are commonly identified from a wide range of freshwater and marine organisms, such as cyanobacteria, marine algae, and seaweed [2, 3]. To date, MAAs have been identified from 572 species of marine macroalgae [4]. In addition, some marine vertebrates and invertebrates, such as medaka fish, *Antarctic krill*, and scallops, acquire MAAs from dietary marine algae [5–7]. MAAs are low-molecular-weight (generally less than 400 Da) and water-soluble molecules that can absorb UV radiation (UVR). MAAs consist of a cyclohexenimine ring conjugated with two amino acid substituents [8].

MAAs can protect cells from solar UVR by absorbing UVR typically between 310 and 340 nm [9]. Moreover, MAAs have diverse functions, including protection from free-radical damage and resistance to several stresses, such as hypersalinity, desiccation, and heat stress [2, 10]. To date, the chemical structures of more than 30 different MAAs as natural metabolites have been revealed [11]. In microalgae, the six most abundant components of MAAs were found to be palythene, palythine, mycosporine-glycine, palythenic acid, porphyra-334, and shinorine [3].

Porphyra-334, a kind of MAA, is a potential natural metabolite that can protect against UVA [12, 13] and has antioxidant activity [14, 15]. Porphyra-334 was initially identified from the red alga, *Porphyra tenera* Kjellman, and its amino acid structure was determined in 1979 [16]. Since then, porphyra-334 has been purified from diverse marine organisms, such as *Bangia atropurpurea* [17], *Nostoc sphaericum* [18], *Porphyra vietnamensis* [19], and *Porphyra yezoensis* [20]. Porphyra-334's protective effect against UVA has been examined in diverse human cells. For instance, porphyra-334 was shown to inhibit UV-induced apoptosis in human immortalized keratinocyte (HaCaT) cells by attenuation of the caspase pathway [21]. Porphyra-334 extracted from *Porphyra yezoensis* induced photoaging in human skin fibroblasts in response to UVA irradiation by suppressing reactive oxygen species (ROS) production and expression of nuclear factor-*κ*B (NF-*κ*B) but activating the NF-E2-related factor 2 (Nrf2) signaling pathways [20, 22]. A previous study conducted transcriptome analysis of human keratinocyte cells in the presence of porphyra-334 exposed to UVR using cDNA and miRNAs microarray [23]. Furthermore, our recent study demonstrated that porphyra-334 efficiently promotes cell reprogramming by activating both mesenchymal to epithelial transition (MET) and the trimethylation of histone 3 lysine 4 (H3K4me3) [24].

Although porphyra-334 has a wide range of functions, its potential role as a cosmetic material has not been well studied. In this study, we characterized porphyra-334 for the promotion of collagen synthesis by *in vitro* assay and for the reduction of periorbital wrinkling by *in vivo* clinical test. In addition, we established transcriptome profiling of human follicle dermal papilla (HFDP) cells in response to porphyra-334 by RNA-seq to reveal the gene expression network associated with porphyra-334.

## 2. Materials and Methods

### 2.1. Preparation of Porphyra Extract and Porphyra-334

*Porphyra yezoensis* used in this study was purchased from a market. We obtained porphyra extract by heat extraction with 25 kg of *Porphyra yezoensis*, 988 kg of distilled water, 10 kg of butylene glycol, and 2 kg of 1, 2-Hexanediol at 40–50°C for 12 h. Porphyra-334 was purified from the porphyra extract. The content of porphyra-334 in the porphyra extract was found to be 1000 ppm based on HPLC analysis.

### 2.2. Cultivation of Human Cells

Three different types of human cells were used in this study: human Detroit 551 fibroblast cells (ATCC, Manassas, VA, USA), human immortalized keratinocyte (HaCaT) cells (ATCC), and HFDP cells derived from mainly normal human scalp hair follicles (ScienCell, San Diego, CA, USA). Detroit 551 and HaCaT cells were cultivated in Dulbecco's Modified Eagle Medium (DMEM) (Welgene, Gyeongsan-si, Korea) supplemented with 10% Fetal Bovine Serum (FBS) (Thermo Fisher Scientific, Waltham, MA, USA) and penicillin-streptomycin (10,000 U/mL) (Thermo Fisher Scientific) at 37°C with a 5% CO_2_ condition. We cultivated HFDP cells in mesenchymal stem cell medium-basal (MSCM-b) (ScienCell) including 10% FBS and 1X Antibiotics at 37°C in 5% CO_2_.

### 2.3. Evaluation of Cell Metabolic Activity by MTT Assay

We conducted an MTT (3-(4, 5-dimethylthiazol-2-yl)-2, 5-diphenyltetrazolium bromide) assay to measure the effect of porphyra extract and porphyra-334 on the cell viability, proliferation, and cytotoxicity of the three different human cells (Detroit 551, HaCaT, and HFDP cells). We treated Detroit 551 cells with three different concentrations (1, 5, and 10 ppm) of porphyra extract and porphyra-334. HaCaT and HFDP cells were both treated with six different concentrations of porphyra-334 (1, 5, 10, 50, 100, and 200 ppm). Distilled water was used as a control. We incubated Detroit 551, HaCaT, and HFDP cells at a density of 5 × 10^4^ cells per well in a 96-well plate for 24 h. Individual cells were treated with different concentrations of porphyra extract, porphyra-334, and distilled water. After 24 h of treatment, the medium was removed, 4 *μ*L of 5 mg/mL MTT (Sigma-Aldrich, St. Louis, MO, USA) was added, and they were incubated for 4 h. We again removed the medium, added 100 *μ*L of dimethylsulfoxide (DMSO) (Sigma), and let it dissolve for 10 min. We measured the wavelength absorbance at 570 nm using a Thermo Scientific Multiskan GO Microplate Spectrophotometer (Fisher Scientific Ltd., Vantaa, Finland). Cell viability was calculated using the following formula: cell viability (%) = (amount of absorbance of treated cells/amount of absorbance of control cells) *x* 100.

### 2.4. Measurement of Collagen Synthesis

To measure the improvement in collagen synthesis by porphyra extract and porphyra-334, we determined procollagen expression levels using the Procollagen Type I C-Peptide (PIP) EIA kit according to the manufacturer's instructions (Takara Korea Biomedical Inc., Seoul, Korea).

### 2.5. Clinical Evaluation of Efficacy of Cream Containing Porphyra-334 for Improvement of Periorbital Wrinkles

The clinical study for the evaluation of the efficacy of a cream containing porphyra-334 (10 ppm) for the improvement of periorbital wrinkles was carried out by the Skin Research Center (Seoul, Korea) after approval was obtained based on the standard operating procedures of the Skin Research Center IRB. The research proposal (KC-IRB-31) was submitted on September 10, 2019, and accepted on October 05, 2019. All clinical evaluations were carried out according to the guideline for Good Clinical Practice (GCP) by the International Conference on Harmonization.

We selected a total of 23 healthy female volunteers (30 to 65 years old) showing periorbital wrinkles. The test product (porphyra-334) was a cream containing porphyra-334 (10 ppm), and the control product was a cream without porphyra-334. All volunteers applied the test and control products to the eye area after washing their face. The treatment was randomly conducted on the left or right side of volunteers' faces. Our test was a double-blind test in which neither the subjects nor the researchers knew which product was a test sample. The treatment was conducted in the morning and in the evening for 12 weeks. We examined skin and took photographs at three different time points: baseline before use, after 6 weeks, and after 12 weeks. Periorbital wrinkles were measured by a Skin Visiometer SV700 (Courage and Khazaka, Köln, Germany). The images of individual volunteers' periorbital wrinkles were taken using Mark-Vu (PSI Plus, Suwon, Korea). The evaluations of periorbital wrinkling were conducted using three different methods. The first was the measurement of periorbital wrinkles by a Skin Visiometer SV700 based on periorbital wrinkling parameters: R1 (skin roughness), R2 (maximum roughness), R3 (average roughness), R4 (smoothness depth), and R5 (arithmetic average roughness). The second was a self-assessment by volunteers in which the participants filled out questionnaires on their global assessment of the efficacy and their subjective satisfaction. The third was a visual assessment by two dermatologists.

### 2.6. RNA Extraction, Library Preparation, and Next-Generation Sequencing

To establish a transcriptome profile of HFDP cells in response to porphyra-334 treatment, HFDP cells at a density of 1 × 10^6^ cells per well were incubated in a six-well plate for 24 h. Next, we treated HFDP cells with 10 ppm of porphyra-334 (treatment) or distilled water (control) for 24 h. We harvested three different biological replicates for each condition. We extracted total RNA using an RNeasy Mini Kit (Qiagen, Hilden, Germany) according to the manufacturer's instructions. The extracted total RNAs were used for mRNA library preparation using the TruSeq Stranded mRNA LT Sample Prep Kit according to the manufacturer's instructions. We generated six different libraries from two different conditions with three biological replicates. The six libraries were paired-end sequenced by Illumina's NovaSeq 6000 system (Macrogen, Seoul, Korea). We deposited the obtained raw sequence data in the National Center for Biotechnology Information (NCBI) Sequence Read Archive (SRA) database with the following respective accession numbers: SRR12641544—SRR12641549.

### 2.7. Transcriptome Analysis and Gene Ontology Term Enrichment Analysis

Raw sequence reads from each library were trimmed using the PRINSEQ program with a quality score of 20 [25]. Next, clean reads were mapped on the human reference transcripts version GRCh38 (https://www.ncbi.nlm.nih.gov/genome/guide/human/) using the BWA with default parameters (http://bio-bwa.sourceforge.net/) resulting in SAM file format. The SAM files containing mapping information were subjected to the eXpress program (https://pachterlab.github.io/eXpress/manual.html) to calculate the number of reads mapped on individual human transcripts. The comma-separated values (CSV format) containing the number of reads for individual human transcripts were obtained using the GENAVi (https://junkdnalab.shinyapps.io/GENAVi/) program. Normalization was conducted using the DESeq2 package implemented in GENAVi. Differentially expressed genes (DEGs) between treatment and control conditions were identified based on adjusted *p* values of less than 0.01 and log2 converted fold changes of more than 1.

To identify enriched functions in the identified DEGs, we conducted gene ontology (GO) term enrichment analysis using the WEB-based Gene SeT AnaLysis Toolkit (WebGestalt) (http://www.webgestalt.org/) [26]. The identified DEGs were divided into two groups: upregulated genes (39 transcripts) and downregulated genes (43 transcripts). The gene list in each group was subjected to overrepresentation analysis against the GO, KEGG, Panther, Reactome, WikiPathways, and WikiPathways Cancer databases with FDR 0.05 as a cutoff. The overrepresented GO terms were visualized by the directed acyclic graph (DAG) structure according to three categories: biological process (BP), cellular component (CC), and molecular function (MF).

### 2.8. Statistical Analysis

We conducted a one-way ANOVA test to compare the control and test samples. The results are shown as mean and standard deviation (mean ± SEM). The *p* values *p* < 0.05^(^∗^)^, *p* < 0.01^(^∗∗^)^, and *p* < 0.001^(^∗∗∗^)^ were considered statistically significant. For the clinical evaluation of periorbital wrinkling, we conducted two different statistical analyses: paired samples *t-*test and independent *t-*test. *p* values for *t-*test comparisons with baseline values are as follows: ^∗^ indicates *p* < 0.05, ^∗∗^ indicates *p* < 0.01, and ^∗∗∗^ indicates *p* < 0.001. ^∗^*p* values for *t-*test comparisons between values in the test group and the control group are as follows: ^#^ indicates *p* < 0.05. In the case of R5 and visual assessment, statistical analyses were carried out as follows: † indicates *p* < 0.05 by Wilcoxon signed ranks test; ^∗^ indicates *p* < 0.05 by paired samples *t-*test; ^‡^ indicates *p* < 0.05 by Mann–Whitney *U* test; ^†^ indicates *p* values for *t*-test comparisons between values in the test group and the control group; ^†^ indicates *p* < 0.05; ^††^ indicates *p* < 0.01. All statistical tests were declared statistically significant at the 0.05 level. We used IBM SPSS Statistics version 21.0 (SPSS, Chicago, IL, USA) for the statistical analysis.

## 3. Results

### 3.1. Evaluation of Cell Viability and Collagen Formation by Porphyra Extract and Porphyra-334 in Detroit 551 Cells

**Figure 1:**
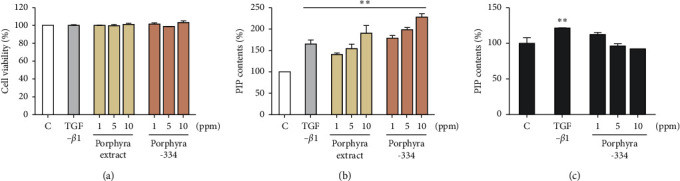
*In vitro* assessment of the effect of different concentrations of porphyra extract and porphyra-334 on cell viability and collagen synthesis. The effect of three different concentrations (1, 5, and 10 ppm) of porphyra extract and porphyra-334 on the cell viability of Detroit 551 cells was examined by MTT assay (a). C indicates control cells treated with distilled water. (b) PIP content was measured in Detroit 551 cells after treatment with different concentrations of porphyra extract and porphyra-334. TGF-*β*1, which is known to promote collagen formation, cell proliferation, and the differentiation of dermal fibroblasts, was used as a positive control. (c) PIP content was measured in HFDP cells after treatment with different concentrations of porphyra-334. For the comparisons between the positive sample (TGF-*β*1) and the treatment group, we conducted independent *t*-tests. ^∗∗^*p* < 0.01.

We examined the effect of porphyra extract derived from *Porphyra yezoensis* and porphyra-334 on the cell viability and proliferation of Detroit 551 cells derived from human skin fibroblast cells. We used three different concentrations (1, 5, and 10 ppm) of both porphyra extract and porphyra-334. Cell viability was slightly increased after the application of 1 and 10 ppm of both porphyra extract and porphyra-334; however, the application of 5 ppm of porphyra extract and porphyra-334 resulted in a slight reduction of viability (Figure 1(a)). The MTT assays showed that the application of 1 and 10 ppm of porphyra extract and porphyra-334 resulted in no cellular toxicity in Detroit 551 cells.

### 3.2. Evaluation of Collagen Formation by Porphyra Extract and Porphyra-334 in Detroit 551 Cells

We measured the effect of porphyra extract and porphyra-334 on collagen formation ability in Detroit 551 cells (Figure 1(b)). Again, Detroit 551 cells were treated with three different concentrations (1, 5, and 10 ppm) of porphyra extract and porphyra-334. Transforming growth factor-beta 1 (TGF-*β*1), which is known to promote collagen type 1 production, was used as a positive control [27]. As compared to the control (distilled water), PIP content was increased 144% by TGF-*β*1 treatment (Figure 1(b)). After the application of porphyra extract and porphyra-334, PIP content was increased as the concentration of porphyra extract and porphyra-334 increased. For example, PIP content was increased by 121% and 130% after the application of 1 ppm of porphyra extract and porphyra-334, respectively. PIP content was increased by 147% and 154% after the application of 10 ppm of porphyra extract and porphyra-334, respectively. The application of 5 ppm of porphyra extract and porphyra-334 resulted in similar PIP content to TGF-*β*1. The statistical analysis demonstrated that both porphyra extract and porphyra-334 significantly promoted collagen synthesis (*p*=0.01). Next, we examined PIP content in HFDP cells after treatment with three different concentrations (1, 5, and 10 ppm) of porphyra-334 (Figure 1(c)). PIP content in HFDP cells was increased by 121% by TGF-*β*1 (*p*=0.01). After the application of porphyra-334 in HFDP cells, PIP content was decreased as the concentration of porphyra-334 increased. For instance, PIP content in HFDP cells was increased 112% by the treatment of 1 ppm porphyra-334. By contrast, PIP content in HFDP cells was decreased by 5 ppm (96%) and 10 ppm (92%) of porphyra-334. In addition, we examined PIP content in HaCaT cells after treatment with different concentrations of porphyra-334. However, PIP was not expressed in HaCaT cells.

### 3.3. Effect of Porphyra-334 on Cell Viability in HaCaT and HFDP Cells

**Figure 2:**
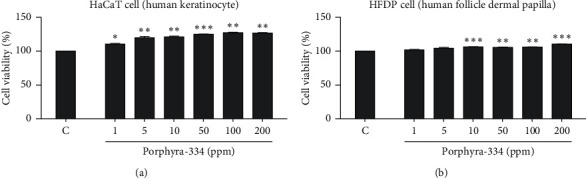
*In vitro* assessment of the effect of different concentrations of porphyra-334 on cell viability in HaCaT and HFDP cells. The effect of six different concentrations (1, 5, 10, 50, 100, and 200 ppm) of porphyra-334 on cell viability was examined in HaCaT (a) and HFDP (b) cells by MTT assay. Control (c) indicates cells treated with distilled water. For the comparisons between the positive sample (TGF-*β*1) and the treatment group, we conducted independent *t*-tests. ^*∗*^*p* < 0.05, ^*∗∗*^*p* < 0.01, and ^*∗∗∗*^*p* < 0.001.

It is important to examine the effect of porphyra-334 on cell viability and proliferation in different human cell lines and to determine whether increasing the concentration of porphyra-334 affects cell viability and proliferation. For that, we examined the effect of six different concentrations (1–200 ppm) of porphyra-334 on cell viability in two different human cell lines: human keratinocyte (HaCaT) and HFDP cells (Figures 2(a) and 2(b)). As compared to the control treated with distilled water, the MTT assay showed that the cell viability of the HaCaT cells increased as the concentration of porphyra-334 increased (Figure 2(a)). For example, the application of 1 ppm of porphyra-334 resulted in 110.33% cell viability, while 200 ppm of porphyra-334 resulted in 126.68% cell viability. The statistical analyses support that all six concentrations of porphyra-334 increased cell viability as compared to the control. In the case of HFDP, the treatment of HFDP cells with porphyra-334 resulted in an increase of cell viability (101.79% (1 ppm) to 110.26% (200 ppm)) as compared to the control (Figure 2(b)). In particular, 10 ppm (*p*=0.001), 50 ppm (*p*=0.01), 100 ppm (*p*=0.01), and 200 ppm (*p*=0.001) of porphyra-334 increased the cell viability of HFDP cells. However, the increase of cell viability by porphyra-334 in HaCaT cells was much higher than that in HFDP cells.

### 3.4. Clinical Evaluation of Porphyra-334 for Reduction of Periorbital Wrinkling

**Figure 3:**
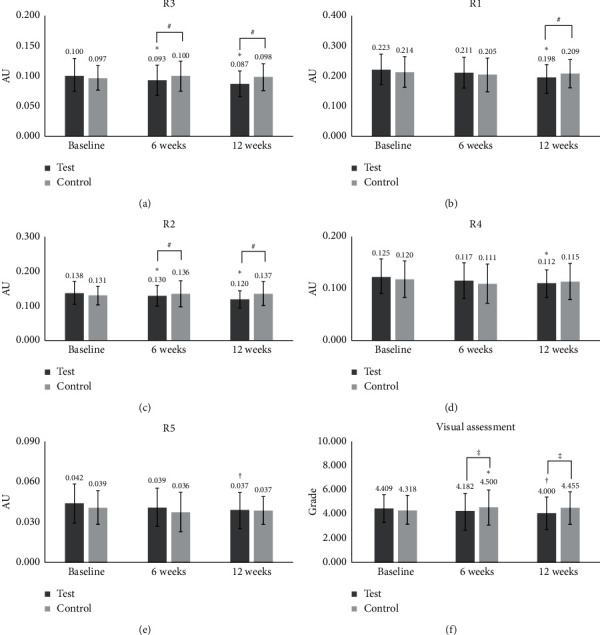
Clinical tests of porphyra-334 for improving periorbital wrinkles. Test (containing porphyra-334 (10 ppm)) and control (without porphyra-334) products were used. Six periorbital wrinkling parameters ((a) R3, average roughness; (b) R1, skin roughness; (c) R2, maximum roughness; (d) R4, smoothness depth; (e) R5, arithmetic average roughness, and (f) visual assessment) were measured for 22 participants at three different time points: baseline, 6 weeks (6 (W), and 12 weeks (12 (W). For the comparisons with baseline, we conducted paired samples *t*-tests. ^∗^ indicates *p* < 0.05. For the comparisons between the test group and the control group, we used independent *t*-tests. ^#^ indicates *p* < 0.05. For the visual assessment, we used the Wilcoxon signed ranks test and Mann–Whitney *U* test. ^†^*p* < 0.05. ^‡^*p* < 0.05. AU = arbitrary unit.

Next, we examined the possible efficacy of porphyra-334 for the reduction of periorbital wrinkling. For that, we prepared a test product (cream containing 10 ppm of porphyra-334) and a control product (cream without porphyra-334). A total of 23 healthy female participants showing periorbital wrinkles were treated with test and control products. Using a Skin Visiometer SV700, we measured periorbital wrinkling according to five parameters: R1 (skin roughness), R2 (maximum roughness), R3 (average roughness), R4 (smoothness depth), and R5 (arithmetic average roughness). In R3 (average roughness), the periorbital wrinkling in the test group was significantly lower at 6 weeks (*p* < 0.001) and 12 weeks (*p* < 0.001) than at baseline (Table 1 and Figure 3(a)). The reduction of periorbital wrinkling was 1.85 times higher at 12 weeks (−12.798%) as compared to 6 weeks (−6.885%). We did not see any difference at 6 weeks or 12 weeks as compared to baseline in the control group. In comparisons between groups, average roughness values were significantly higher in the test group than those in the control group at 6 weeks and 12 weeks (*p*=0.003 and *p* ≤ 0.001, respectively) (Table 1 and Figure 3(a)).

In the case of R1 (skin roughness), there was no significant difference at 6 weeks or 12 weeks as compared to baseline in the test group (Table 1 and Figure 3(b)). In the control group, skin roughness values were significantly reduced only at 6 weeks (*p*=0.001) as compared to baseline. There was a significant difference in R1 (*p*=0.047) between the test group and the control group only at 12 weeks (Table 1 and Figure 3(b)).

For the measurement of R2 (maximum roughness), we found a significant reduction at both 6 weeks (*p*=0.007) and 12 weeks (*p* ≤ 0.001) as compared to baseline in the test group (Table 1 and Figure 3(c)). However, there was no difference in maximum roughness at either time point as compared to baseline in the control group (Table 1 and Figure 3(c)). In addition, we found that there were significant differences in maximum roughness values between the test and the control groups at 6 weeks (*p*=0.040) and 12 weeks (*p* ≤ 0.001).

In the case of R4 (smoothness depth), there was a significant reduction only at 12 weeks (−8.037%) (*p*=0.006) as compared to baseline in the test group (Table 1 and Figure 3(d)). For the R5 (arithmetic average roughness) measurement, the test group at 12 weeks (−11.071%) (*p*=0.008) showed a strong reduction as compared to baseline (Table 1 and Figure 3(e)). There was no difference between the groups for smoothness depth (Table 1 and Figure 3(e)).

According to a visual assessment of periorbital wrinkling, there was a significant reduction at 12 weeks (−10.952%) (*p*=0.003) in the test group, while in the control group, the periorbital wrinkling was increased at 6 weeks (3.225%) (*p*=0.042) (Table 1 and Figure 3(f)). There were significant differences in periorbital wrinkling between the two groups at 6 weeks (*p*=0.007) and 12 weeks (*p* ≤ 0.001) (Table 1 and Figure 3(f)).

Six periorbital wrinkling parameters (R1, skin roughness; R2, maximum roughness; R3, average roughness; R4, smoothness depth; R5, arithmetic average roughness, and visual assessment) were measured at three different time points: baseline, 6 weeks (6 W), and 12 weeks (12 W). In the case of R1–R4, two different statistical analyses (paired samples *t-*test ^∗^ and independent *t-*test ^#^) were carried out. ^∗^*p* values for *t*-test comparisons with baseline values; ^∗^ indicates *p* < 0.05; ^∗∗^ indicates *p* < 0.01; ^∗∗∗^ indicates *p* < 0.001. ^∗^*p* values for *t*-test comparisons between values in the test group and the control group; ^#^ indicates *p* < 0.05. In the case of R5 and visual assessment, statistical analyses were carried out as follows. ^†^: *p* < 0.05 by Wilcoxon signed ranks test. ^∗^: *p* < 0.05 by paired samples *t-*test. ^‡^: *p* < 0.05 by Mann–Whitney *U* test. ^†^*p* values for *t*-test comparisons between values in the test group and the control group; ^†^*p* < 0.05; ^††^*p* < 0.01. Change (%) = [(value after treatment–value before treatment)/value before treatment]x100.

Three biological samples from control (distilled water) and treatment (10 ppm of porphyra-334) HFDP cells were used for library preparation followed by paired-end sequencing with NovaSeq 6000.

### 3.5. Transcriptome Profiling of HFDP Cells in response to Porphyra-334 Treatment by RNA-Seq

Although porphyra-334 has a wide range of important roles, the genomewide expression profile for human genes in response to porphyra-334 treatment has not been established. In this study, we performed RNA-seq to establish transcriptome profiling in response to porphyra-334 treatment. The MTT assays demonstrated that the cell viability and proliferation in HFDP cells treated with porphyra-334 were the lowest among the three different human cell lines. However, porphyra-334 treatment in HFDP cells might result in dramatic changes in the transcriptome. Moreover, it might be of interest to examine the possible role of porphyra-334 in the promotion of hair follicle development by RNA-seq. We selected 10 ppm of porphyra-334, which was shown to increase cell viability with strong statistical significance for RNA-seq.

**Figure 4:**
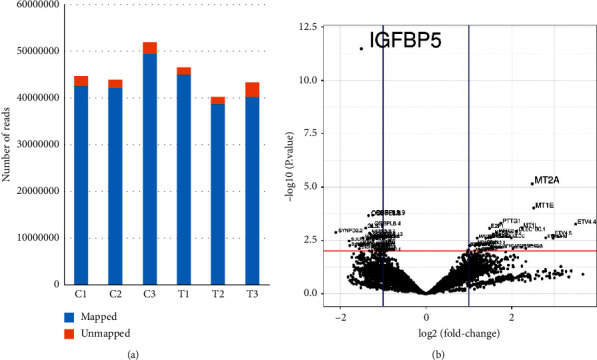
Mapping results and visualization of DEGs. (a) Proportion of mapped (blue) and unmapped (orange) reads on human reference transcriptome. (b) Volcano plot displaying the distribution of log10 (padj) and log2 (FC) for all expressed genes. Padj and FC indicate the adjusted *p* value and fold change, respectively. DEGs are indicated by individual transcript names.

HFDP cells were treated with 10 ppm of porphyra-334 (treatment) or distilled water (control). After 24 h, we harvested the cells and extracted total RNAs. The extracted total RNAs were used for mRNA library preparation as described previously. Three independent biological replicates for each condition were used for library preparation. A total of six libraries were paired-end sequenced using the NovaSeq 6000 system (Table 2). The number of sequence reads ranged from 40, 123, 294 (T2) to 51, 733, 698 (C3) (Figure 4(a)). The low-quality sequences from the obtained raw data were trimmed with a quality score of 20. The ratio of bases with Phred quality scores of more than 20 ranged from 98.46% to 98.61%, demonstrating the high quality of RNA-seq (Table 2). The proportion of mapped reads on the human transcriptome containing 159,998 transcripts ranged from 92.79% (T3) to 96.21% (T1) (Figure 4(a)). The number of mapped reads from each library was used for normalization using the DESeq2 method implemented in GENAVi (https://junkdnalab.shinyapps.io/GENAVi/). An adjusted *p* value less than 0.01 and a fold change of more than 2 were used for thresholds to identify DEGs (Figure 4(b)). Finally, we identified 27 upregulated genes (39 transcripts) and 16 downregulated genes (43 transcripts) (Tables 3 and 4).

### 3.6. Identification of DEGs in HFDP Cells in response to Porphyra-334 Treatment

**Figure 5:**
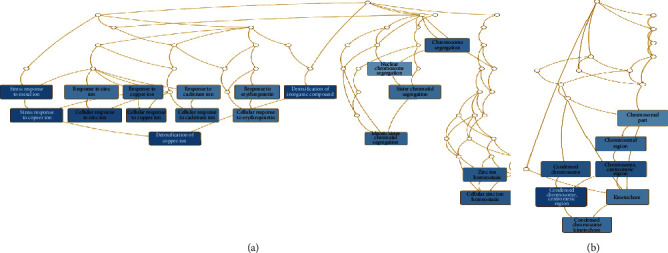
Hierarchical structure of identified enriched GO terms for upregulated genes in response to porphyra-334 treatment. DAGs visualize the hierarchical structure of identified enriched GO terms for upregulated genes in response to porphyra-334 treatment according to biological process (a) and cellular component (b). Each GO term is indicated by a different box color based on *p* value. Detailed information on identified GO terms can be found in Table S1.

Of the identified DEGs, there were several different transcripts transcribed from a single gene. For example, three BIRC5 transcripts, three ETV1 transcripts, and four PTTG1 transcripts were upregulated (Table 3), while 10 IL1R1 transcripts, 12 OSBPL8 transcripts, and three SLC6A6 transcripts were downregulated by porphyra-334 (Table 4). To find enriched functions in the identified DEGs, we carried out GO enrichment analysis using the WebGestalt program. According to biological function for the 27 upregulated genes, GO terms were associated with cellular response to different metal ions (GO:0097501), such as stress response to copper ion (GO:1990169), cellular response to zinc ion (GO:0071294), and cellular response to cadmium ion (GO:0071276) (Table S1 and Figure 5(a)). Three genes (MT1E, MT1X, and MT2A), which are members of the metallothionein (MT) family, were involved in the detoxification of the inorganic compound (GO:0061687). In addition, four upregulated genes (MAD2L1, NCAPG, NEK2, and PTTG1) were involved in mitotic sister chromatid segregation (GO:0000070), sister chromatid segregation (GO:0000819), and nuclear chromosome segregation (GO:0098813). Two upregulated genes, MT1X and MT2A, were involved in the cellular response to erythropoietin (GO:0036018). According to the cellular component, several GO terms associated with chromosomal part were enriched (Table S1 and Figure 5(b)): chromosome centromeric region (GO:0000775), condensed chromosome (GO:0000793), and kinetochore (GO:0000776). In particular, four genes (BIRC5, MAD2L1, NCAPG, and NEK2) were associated with the chromosomal region (GO:0098687). Additionally, we identified an enriched metabolic pathway (mineral absorption (hsa04978)) according to the KEGG database. Four genes (MT1E, MT1L, MT1X, and MT2A) were involved in zinc homeostasis (WP3529) and copper homeostasis (WP3286) according to WikiPathways.

### 3.7. Gene Expression Profile of Hair Cycle and Hair Follicle Structure-Associated Genes

We examined the expression of selected genes associated with the hair cycle based on the previous study (Table 5). Out of 27 hair cycle-associated genes, *BMP2* encoding bone morphogenetic protein 2, *CYP1A1* encoding cytochrome P450 family 1 subfamily A member 1, *CYP27B1* encoding cytochrome P450 family 1 subfamily B member 1, and *WNT5A* encoding Wnt family member 5A were significantly upregulated by porphyra-334 treatment. Next, we examined the expression of 10 selected genes associated with the hair follicle structure (Table S2). Several keratin genes, such as *KRT33 B*, *KRT34*, and *KRT79*, were upregulated, while the *KRT16* gene was downregulated by porphyra-334 treatment. In addition, *CDH3* encoding P-cadherin 3 and *TCHH* encoding trichohyalin were upregulated. Out of nine selected genes associated with the epidermal structure, *DSG1* encoding desmoglein (Dsg) 1 and *KRT1* encoding keratin 1 protein were strongly upregulated, while *DSC1*, *DSP*, *FLG*, and *PLEC* genes were downregulated (Table S2). Of 10 selected genes associated with stem cells, *CD34*, *KRT15*, and *SOX9* were significantly upregulated (Table S2).

## 4. Discussion

Human aging is a natural process accompanied by a decline in physiological and psychological functions. Intrinsic aging and photoaging cause skin aging, with diverse signs such as changes in skin tone, elasticity, vasodilation, and wrinkles. In particular, wrinkles can be easily affected by age and various stresses, including UV irradiation, which causes skin inflammation, pigmentation, and skin cancer. The photoaging induced by UV irradiation reduces collagen formation and induces the expression of matrix metalloproteinases (MMPs), resulting in wrinkle formation [28]. MMPs degrade extracellular matrix (ECM) components, such as collagen, elastin, and gelatin. Of the known MMPs, *MMP1* encodes interstitial collagenase, specifically degrading the collagen triple helix, while *MMP2* encodes gelatinase A, degrading denatured collagen, gelatin, and elastin [29]. About 30% of the human body's proteins are collagen. Bundles of collagen molecules referred to as collagen fibers are the largest components of the ECM, providing the strength and elasticity of human tissues and organs [30]. Mature type I collagen fibers are synthesized by type I procollagen molecules, which are composed of two pro-alpha-1 chains (COL1A1) and one pro-alpha-2 chain (COL1A2) [31].

Porphyra-334 treatment led to a dramatic increase of collagen synthesis in Detroit 551 cells. In particular, the effect of 1 ppm of porphyra-334 on collagen synthesis was higher than that of TGF-*β*1 used as a positive control, which promotes the collagen formation of dermal fibroblasts [27]. Although porphyra extract containing porphyra-334 and other MAAs also promoted collagen synthesis, the effect of purified porphyra-334 on collagen synthesis was much better. Moreover, as the concentration of porphyra-334 was increased, the effect on collagen synthesis increased. Thus, these results indicate that porphyra-334 might be a potential molecule for the promotion of collagen synthesis. By contrast, PIP contents in HFDP cells were decreased after different concentrations of porphyra-334 treatment. Moreover, PIP was not expressed in HaCaT cells. These results suggest the cell-specific expression of PIP.

Our study demonstrated that the effects of porphyra-334 on cell viability and proliferation were dependent on the cell type, and the increase of cell viability by porphyra-334 was the highest in HaCaT cells among the three tested cell types. Porphyra-334 also statistically significantly increased the cell viability of HFDP cells; however, cell viability was not significantly changed in Detroit 551 cells. Of the different concentrations of porphyra-334, the application of 10 ppm of porphyra-334 showed a significant increase in cell viability, while 1 and 5 ppm of porphyra-334 did not show any increase of cell viability. This result suggests that the optimal concentration of porphyra-334 should be applied for cosmetic purposes.

The *in vivo* clinical test with 22 participants demonstrated the effect of porphyra-334 (10 ppm) on the reduction of periorbital wrinkles. Of the six examined parameters of periorbital wrinkles, three (R3 (average roughness), R2 (maximum roughness), and visual assessment)) were significantly different between the treatment and control groups at both 6 and 12 weeks. Our recent study showed the improvement of periorbital wrinkles by Leontopodium Alpinum callus culture extract (LACCE) [32]. Similarly, a recent study using a cream containing low-molecular-weight heparan sulfate (LMW‐HS) and a mixture of four different naturally derived plant extracts showed the improvement of periorbital wrinkles [33]. However, we showed the usefulness of porphyra-334 as a single molecule for the reduction of periorbital wrinkles as compared to the other two studies.

Next-generation sequencing (NGS) is currently used for a wide range of molecular studies. RNA-seq based on the NGS technique can reveal the presence and quantity of RNA in a specific biological sample. RNA-seq is particularly useful to analyze the genomewide transcriptome change by a specific treatment. In this study, we carried out transcriptome profiling of HFDP cells, which were isolated from the hair papilla of normal human scalp hair follicles in response to porphyra-334 treatment. It was of interest to examine the effects of porphyra-334 in different human cell lines. However, due to financial constraints, we selected HFDP cells to study the potential roles of porphyra-334 in the control of hair production and the hair growth cycle. RNA-seq identified several DEGs that were up- or downregulated by porphyra-334 treatment. Of the identified DEGs, there were multiple transcripts (isoforms) derived from the same gene in our study, and the isoforms showed similar expression patterns. This result suggests that the isoforms of the identified DEGs in this study had similar expression patterns, at least in HFDP cells. On the other hand, several other studies have reported that the differential expression of isoforms of a gene in different organs, tissues, cells, and developmental stages occurs very often [34, 35].

Gene enrichment analysis revealed that four genes *MT1E*, *MT1L*, *MT1X*, and *MT2A* encoding MTs were upregulated by porphyra-334 treatment. MTs are ubiquitous small cysteine-rich proteins containing two binding domains, *α* and *β*, which enable them to bind to several heavy metals, such as cadmium, copper, and zinc [36]. Due to their unique structural characteristics, MTs function in metal homeostasis [37] and protection against oxidative stresses [38]. Free radicals such as ROS and reactive nitrogen species (RNS) are generated by UV, X-ray, and gamma radiation, and they are responsible for aging and several human diseases, including DNA and cell damage and diverse cancers [39–41]. The functional roles of MTs as free-radical scavengers against ROS and RNS have been previously demonstrated [42, 43]. In addition, MTs are involved in the inhibition of apoptosis [44] and carcinogenesis [45]. In humans, MTs have four different isoforms (MT1–MT4), and MT1 consists of eight known functional isoforms [45]. Based on the results, the overexpression of three MT1 isoforms and one MT2 isoform in the HFDP cells by porphyra-334 strongly suggests the potential antiaging effect of porphyra-334 as an antioxidant cooperative with MTs in human cells. It might be of interest to examine the possible interaction of MTs with porphyra-334 in the near future.

Of the upregulated genes, *BIRC5*, also known as survivin, is a member of the inhibitor of apoptosis protein (IAP) family and functions in the control of cell proliferation and inhibition of apoptosis [46]. A previous study showed that survivin is expressed in the proliferating keratinocytes of the hair matrix and outer root sheath of human anagen hair follicles [47]. UV-B induced the expression of survivin in human keratinocytes and mouse skin, suggesting the possible role of survivin in response to UV-B [48]. Furthermore, Wnt/*β*-catenin signaling might control the expression of survivin in anagen hair follicles [47]. A previous study reported that cyclin-dependent kinase 1 (*CDK1*) is also upregulated in HDPC cells in response to platelet-rich plasma (PRP), which is plasma enriched with a higher proportion of platelets [49]. Thus, our results indicate that the overexpression of *BIRC5* and *CDK1* in HFDP cells by porphyra-334 might be associated with the promotion of hair follicle growth. However, further studies should be conducted to confirm our hypothesis.

Some genes upregulated by porphyra-334 are involved in carcinogenesis. For example, there have been reports of the promotion of colorectal cancer progression by cell division cycle-associated 5 (CDCA5) [50] and the functional role of E26 transformation-specific (ETS) variant 4 (ETV4) in pancreatic cancer [51]. The pathological significance of MAD2L1 in breast cancer [52]; the metastasis promotion of PITPNC1 by melanoma, breast cancer, and colon cancer cells [53]; and the suppression of breast tumors by RIN1 [54] have also been reported.

Two upregulated genes, E2F1 and FOXL1, were associated with cell proliferation. Moreover, the functional roles of E2F1 for the promotion of apoptosis and suppression of proliferation have been reported [55]. FOXL1 is a member of the forkhead box (Fox) transcription factor family, and it is involved in the regulation of epithelial cell proliferation in the gastrointestinal tract and the inhibition of growth and invasion in human pancreatic cancer cells [56].

One interesting finding was that three upregulated genes, *BIRC5*, *MAD2L1*, and *NEK2*, encoded proteins that are components of kinetochores. Kinetochores are large protein complexes assembled on the centromeric region of the chromosomes, and they mediate spindle–microtubule attachment and control the movement of chromosomes during mitosis and meiosis [57]. Moreover, porphyra-334 upregulated the expression of four genes, *MAD2L1*, *NCAPG*, *NEK2*, and *PTTG1*, required for nuclear chromosome segregation. These results suggest possible implications of porphyra-334 for chromosome segregation, which is essential for humans to maintain genome stability by mitotic and meiotic divisions [58].

Moreover, we also found that two genes, *ARHGAP22* and *PTTG1*, were associated with cell structure and movement. For instance, ARHGAP22 is a member of the Rho family of small GTPases that regulate morphogenesis, polarity, movement, and cell division [59]. A previous study showed that ARHGAP22 localizes at endosomes and functions in the regulation of the actin cytoskeleton [60]. Pituitary tumor transforming gene 1 (PTTG1), also known as securin, has a wide range of functions, such as inhibition of sister chromatid separation and regulation of microtubule nucleation and cell migration [61].

Among the downregulated genes, many isoforms of *IL1R1*, *OSBPL8*, and *SLC6A6* were coregulated. *IL1R1* encodes interleukin 1 receptor, also called CD121a (Cluster of Differentiation 121a), which is a receptor for interleukin 1 alpha (IL1A), interleukin 1 beta (IL1B), and interleukin 1 receptor antagonist (IL1RA) [62]. IL1R1 transmits the signal of interleukin 1 involved in several cytokine-induced immune and inflammatory responses [62]. The downregulation of IL1R1 by porphyra-334 indicates that porphyra-334 might not be harmful to HFDP cells. *OSBPL8* encoding SBP-related protein 8 (ORP8) is an endoplasmic reticulum sterol sensor functioning in cellular lipid metabolism. A previous study reported that the deficiency of the *OSBPL8* gene in mice resulted in the induction of high-density lipoproteins [63]. It has been reported that SLC6A6 promotes the survival and multidrug resistance of colorectal cancer [64]. The potential roles of OSBPL8 and SLC6A6 associated with porphyra-334 should be further elucidated.

Based on RNA-seq data, we examined the expression of selected genes associated with the hair follicle cycle and structure. As we expected, several keratin genes, such as *Keratin 1, Keratin 10, Keratin 15, Keratin 17, Keratin 33B, Keratin 34*, and *Keratin 79*, were upregulated by porphyra-334, suggesting possible implications of porphyra-334 for the epidermal structure and hair follicle structure. In particular, the expression of *CHD3* encoding P-cadherin was upregulated by porphyra-334. It is known that P-cadherin plays an important role in human hair growth and cycling by regulating canonical Wnt signaling and inhibiting the expression of TGFB2 [65]. Similarly, the expression of *WNT3* and *WNT5A*, which are members of the Wnt family, was upregulated by porphyra-334. However, porphyra-334 upregulated the expression of TGFB2 in our study. The detailed mechanism associated with Wnt signaling and TGFB2 expression regulated by porphyra-334 should be further elucidated. Dsgs are calcium-dependent transmembrane glycoproteins and are involved in keratinization, anchorage of the hair, and hypotrichosis [66]. Of the three DSG genes, *DSG1* and *DSG3* were upregulated, while DSG2 was downregulated. This result is consistent with the previous result showing that the expression of DSG1 and DSG3 was overlapped in the companion layer [67].

Although gene expression profiling of human keratinocyte cells in response to porphyra-334 has been reported previously [23], it was difficult for us to compare the previous results with our results due to differences in human cell lines, concentrations of porphyra-334, UVR treatments, and technical approaches such as microarray and RNA-seq. Therefore, it might be of interest to examine porphyra-334 effects in different human cell lines with or without UVR by RNA-seq for a detailed understanding of the functional roles of porphyra-334.

## 5. Conclusion

In this study, we examined the potential uses of porphyra-334 as an antiaging agent, including the promotion of collagen formation, improvement of periorbital wrinkles, and promotion of cell proliferation, in three different human cell lines. Furthermore, the RNA-seq results confirmed the functional role of porphyra-334 as an antioxidant by upregulating the expression of several genes for MT. Moreover, porphyra-334 induced the expression of genes involved in nuclear chromosome segregation and the encoding of components of kinetochores. In addition, the upregulation of several genes, including many keratin genes associated with the hair follicle cycle, the hair follicle structure, the epidermal structure, and stem cells, suggests the potential role of porphyra-334 for hair follicle growth and maintenance. Taken together, we provided several new pieces of evidence of porphyra-334 as a potential antiaging cosmetic material and elucidated the expression network in HFDP cells in response to porphyra-334.

## Figures and Tables

**Table 1:** Measurement of improvement of periorbital wrinkling according to six parameters.

Evaluation parameter	Time point	Test group			Control group			Test/Control
		Mean ± SD	Change (%)	*p* value †	Mean ± SD	Change (%)	*p* value †	*p* value
R1	Baseline	0.223 ± 0.050	—	—	0.214 ± 0.051	—	—	—
	6 W	0.211 ± 0.052	−5	0.1	0.205 ± 0.054	−3	≤0.001^∗^	0.7
	12 W	0.198 ± 0.040	−10	0.2	0.209 ± 0.048	−1	0.5	0.047^#^

R2	Baseline	0.138 ± 0.033	—	—	0.131 ± 0.027	—	—	—
	6 W	0.130 ± 0.030	−6	0.007^∗^	0.136 ± 0.037	3.3	0.3	0.040^#^
	12 W	0.120 ± 0.024	−12	≤0.001^∗^	0.137 ± .035	4.7	0.2	≤0.001^#^

R3	Baseline	0.100 ± 0.025	—	—	0.097 ± 0.020	—	—	—
	6 W	0.093 ± 0.025	−7	≤0.001^∗^	0.100 ± 0.025	2.9	0.2	0.003^#^
	12 W	0.087 ± 0.021	−13	≤0.001^∗^	0.098 ± 0.022	1.1	0.7	≤0.001^#^

R4	Baseline	0.125 ± 0.034	—	—	0.120 ± 0.035	—	—	—
	6 W	0.117 ± 0.034	−5	0.1	0.111 ± 0.037	−7	0.1	0.8
	12 W	0.112 ± 0.026	−8	0.006^∗^	0.115 ± 0.035	−3	0.3	0.4

R5	Baseline	0.042 ± 0.014	—	—	0.039 ± 0.012	—	—	—
	6 W	0.039 ± 0.014	−7	0.1	0.036 ± 0.014	−7	0.1	1
	12 W	0.037 ± 0.013	−11	0.008^†^	0.037 ± 0.010	−1	0.3	0.4

Visual assessment	Baseline	4.409 ± 1.141	—	—	4.318 ± 1.171	—	—	—
	6 W	4.182 ± 1.532	−8	0.1	4.500 ± 1.439	3.2	0.042^∗^	0.007^‡^
	12 W	4.000 ± 1.345	−11	0.003^†^	4.455 ± 1.371	2.5	0.1	≤0.001^‡^

**Table 2:** Summary of raw data of six different libraries used for RNA-seq.

Library	Description	Total read bases (bp)	GC (%)	Q20 (%)	Accession no.
C1	Control replicate 1	4, 499, 074, 492	50.97	98.46	SRR12641546
C2	Control replicate 2	4, 422, 011, 694	50.65	98.55	SRR12641545
C3	Control replicate 3	5, 225, 103, 498	50	98.55	SRR12641544
T1	Treatment replicate 1	4, 681, 863, 888	48.21	98.61	SRR12641549
T2	Treatment replicate 2	4, 052, 452, 694	50.08	98.57	SRR12641548
T3	Treatment replicate 3	4, 354, 746, 704	50.08	98.58	SRR12641547

**Table 3:** Twenty-seven identified genes (39 transcripts) in HFDP cells upregulated by porphyra-344 treatment.

Transcripts	log2FC	Padj	baseMean	Function
ARHGAP22.11	1.755275	0.006663	76.14885	Rho GTPase activating protein 22
BIRC5	1.14606	0.00556	202.0075	Baculoviral IAP repeat containing 5
BIRC5.3	1.007038	0.005512	275.2747	Baculoviral IAP repeat containing 5
BIRC5.4	1.147806	0.005512	238.167	Baculoviral IAP repeat containing 5
CDCA5.8	1.030194	0.005554	329.4237	Cell division cycle-associated 5
CDK1.1	1.385413	0.004592	278.7067	Cyclin-dependent kinase 1
CDK1.2	1.416186	0.007478	332.39	Cyclin-dependent kinase 1
CLEC18C.1	2.110853	0.001172	137.6678	C-type lectin domain family 18 member C
CSTF3.2	1.719642	0.001671	126.3606	Cleavage stimulation factor subunit 3
E2F1	1.483035	8.70*E* − 04	318.3527	E2F transcription factor 1
ETV4.1	2.798138	0.002372	87.71273	ETS variant transcription factor 4
ETV4.4	3.493913	5.53*E* − 04	78.42156	ETS variant transcription factor 4
ETV4.5	2.97535	0.001792	76.19139	ETS variant transcription factor 4
FOXL1	1.381458	0.009339	824.6779	Forkhead box L1
GLDC	1.994842	0.002396	317.7404	*Glycine* decarboxylase
MAD2L1	1.197541	0.002461	655.8934	Mitotic arrest deficient 2 like 1
MAFF	1.468844	0.007739	443.0851	MAF bzip transcription factor F
MLPH.3	1.133679	0.009733	169.9009	Melanophilin
MT1E	2.516623	9.64*E* − 05	1380.143	Metallothionein 1E
MT1L	2.242355	8.01*E* − 04	1228.073	Metallothionein 1L
MT1X	2.03042	0.007739	520.5107	Metallothionein 1X
MT2A	2.480677	7.07*E* − 06	38795.66	Metallothionein 2A
NCAPG	1.240566	0.005512	185.8562	Non-SMC condensin II complex subunit G2
NCAPG.2	1.162677	0.004724	174.1935	Non-SMC condensin II complex subunit G2
NEFM	2.973343	0.002461	84.93233	Neurofilament medium
NEK2	1.575009	0.001569	163.8964	NIMA related kinase 2
NEK2.2	1.470924	0.004974	127.0178	NIMA related kinase 2
PITPNC1	2.332033	0.007432	171.0734	Phosphatidylinositol transfer protein cytoplasmic 1
PITPNC1.3	2.228156	0.006396	132.9353	Phosphatidylinositol transfer protein cytoplasmic 1
PTTG1	1.749011	4.96*E* − 04	328.4098	PTTG1 regulator of sister chromatid separation
PTTG1.1	1.654699	0.001396	457.2504	PTTG1 regulator of sister chromatid separation
PTTG1.2	1.507915	0.002372	653.1276	PTTG1 regulator of sister chromatid separation
PTTG1.3	1.456208	0.002396	503.3174	PTTG1 regulator of sister chromatid separation
RIN1.1	1.280807	0.008849	188.6867	Ras and Rab interactor 1
RIN1.3	1.28142	0.009534	221.4613	Ras and Rab interactor 1
RNASEH2A	1.397449	0.002461	211.7831	Ribonuclease H2 subunit A
SEC11C.2	1.445373	0.007432	138.2534	Signal peptidase complex subunit
SHISA2	1.314314	0.009339	142.4822	Shisa family member 2
UBE2S.1	1.445318	0.007739	2929.009	Ubiquitin-conjugating enzyme E2 S

Abbreviations: fold change (FC), padj (adjusted *p* value), baseMean (average of normalized count values).

**Table 4:** Sixteen identified genes (43 transcripts) in HFDP cells downregulated by porphyra-334 treatment.

Transcripts	log2FC	Padj	baseMean	Function
TXNRD1.6	−1.04581	0.009608	1816.645	Thioredoxin reductase 1
C1orf198	−1.50685	0.010883	934.3319	Chromosome 1 open reading frame 198
C1orf198.3	−1.55059	0.007739	1214.224	Chromosome 1 open reading frame 198
DHCR7.2	−1.32018	0.009763	333.9369	7-Dehydrocholesterol reductase
GLS.1	−1.40756	8.57*E* − 04	363.402	Glutaminase
IGFBP5	−1.5019	3.25*E* − 12	4046.24	Insulin-like growth factor binding protein 5
IL1R1	−1.07412	0.007432	1560.642	Interleukin 1 receptor type 1
IL1R1.1	−1.15299	0.007432	1595.837	Interleukin 1 receptor type 1
IL1R1.10	−1.10651	0.00554	1810.324	Interleukin 1 receptor type 1
IL1R1.11	−1.10267	0.007796	1826.142	Interleukin 1 receptor type 1
IL1R1.13	−1.1043	0.009733	1556.153	Interleukin 1 receptor type 1
IL1R1.15	−1.08556	0.007432	1626.087	Interleukin 1 receptor type 1
IL1R1.3	−1.11215	0.004592	1519.688	Interleukin 1 receptor type 1
IL1R1.4	−1.14748	0.004604	1575.658	Interleukin 1 receptor type 1
IL1R1.6	−1.06183	0.009772	1515.673	Interleukin 1 receptor type 1
IL1R1.9	−1.07493	0.009772	1442.102	Interleukin 1 receptor type 1
MINDY2.3	−1.14843	0.009763	227.8405	MINDY lysine 48 deubiquitinase 2
OSBPL8	−1.20939	0.005512	928.14	Oxysterol-binding protein like 8
OSBPL8.1	−1.14638	0.002461	944.2872	Oxysterol-binding protein-related protein 8
OSBPL8.10	−1.23771	0.002396	949.3331	Oxysterol-binding protein like 8
OSBPL8.11	−1.37329	0.002848	1047.627	Oxysterol-binding protein like 8
OSBPL8.12	−1.21833	0.001988	977.1096	Oxysterol-binding protein like 8
OSBPL8.3	−1.23386	0.003057	885.1727	Oxysterol-binding protein like 8
OSBPL8.4	−1.26401	6.61*E* − 04	1027.8	Oxysterol-binding protein like 8
OSBPL8.5	−1.22201	0.002396	989.3	Oxysterol-binding protein like 8
OSBPL8.6	−1.31677	0.001419	905.0201	Oxysterol-binding protein like 8
OSBPL8.7	−1.17836	0.00556	888.7467	Oxysterol-binding protein like 8
OSBPL8.8	−1.34186	2.18*E* − 04	898.2552	Oxysterol-binding protein like 8
OSBPL8.9	−1.23957	2.08*E* − 04	875.6725	Oxysterol-binding protein like 8
RSPO3.2	−1.63792	0.00581	95.73672	R-spondin 3
SEMA5A.3	−1.25554	0.009772	120.5946	Semaphorin 5A
SEMA5A.7	−1.24651	0.006395	128.2849	Semaphorin 5A
SLC6A6	−1.32467	0.002396	302.6643	Solute carrier family 6 member 6
SLC6A6.5	−1.2846	0.001671	302.9567	Solute carrier family 6 member 6
SLC6A6.8	−1.30774	0.002461	311.0602	Solute carrier family 6 member 6
SULF1.12	−1.4729	0.002396	122.1485	Sulfatase 1
SULF2	−1.52947	0.00554	89.47182	Sulfatase 2
SULF2.10	−1.79049	0.003386	76.78665	Sulfatase 2
SYNPO2.1	−1.77862	0.005512	663.7285	Synaptopodin 2
SYNPO2.2	−2.09818	0.001336	79.2225	Synaptopodin 2
TXNRD1.3	−1.15061	0.002461	2067.474	Thioredoxin reductase 1
USP53.2	−1.48485	0.005512	223.9112	Ubiquitin specific peptidase 53
USP53.4	−1.5656	0.004986	229.8406	Ubiquitin specific peptidase 53

**Table 5:** Expression of 27 selected hair cycle-associated genes.

Gene ID	Gene function	baseMean	log2FoldChange	*p* value
ADCY7	Activin A receptor type 1	81.85136	−0.53874	0.086809
BMP2	Bone morphogenetic protein 2	1650.067	2.002074	0.027019
BMP7	Bone morphogenetic protein 7	101.2629	0.332366	0.355265
BNC1	Basonuclin 1	22.58606	0.095093	0.866783
BNC2	Basonuclin 2	221.3541	−0.75229	0.008525
CCNA2	Cyclin A2	706.4942	0.597831	0.008916
CYP1A1	Cytochrome P450 family 1 subfamily a member 1	10.70451	2.005344	0.034324
CYP1B1	Cytochrome P450 family 1 subfamily B member 1	26319.37	−0.53917	0.006894
CYP27B1	Cytochrome P450 family 27 subfamily B member 1	11.08607	1.019638	0.172368
DKK3	Dickkopf 3	571.3778	−0.06546	0.780174
FGF18	Fibroblast growth factor 18	57.2295	0.752468	0.10097
FGF5	Fibroblast growth factor 5	272.1485	−0.41585	0.235221
FZD10	Frizzled 10	13.12887	0.504148	0.465732
FZD3	Frizzled 3	29.57211	0.769263	0.099251
HSD17B14	Hydroxysteroid 17-beta dehydrogenase 14	30.8214	−0.36653	0.440247
JAG1	Jagged 1	1349.268	−0.63115	0.012286
MMP7	Matrix metalloproteinase 7	1.600479	0.689856	0.693773
MSX2	Homeobox protein MSX2	83.5912	−0.4325	0.187032
SFRP1	Secreted frizzled-related protein 1	1530.066	−0.03537	0.860464
SFRP2	Secreted frizzled-related protein 2	307.8726	−0.57658	0.042625
SOX10	Sex determining region Y-box 10	2.098739	−0.71721	0.748481
TCF7	Transcription factor 7	4.502755	0.32334	0.754997
TGFB2	Transforming growth factor-beta 2	70.37078	0.566863	0.205471
THBS2	Thrombospondin 2	58576.05	−0.40607	0.018489
VDR	Vitamin *D* receptor	689.8525	−0.58772	0.127502
WNT3	Wnt family member 3	100.6203	0.433119	0.225621
WNT5A	Wnt family member 5A	768.6411	1.30595	0.108462

## Data Availability

Six raw sequence datasets associated with this project were deposited in SRA database under PRJNA663440.

## Supplementary Materials

Supplementary Materials Table S1: enriched GO terms in 27 genes in HFDP cells upregulated by porphyra-334 treatment. Table S2: expression of selected genes involved in hair follicle structure, epidermal structure, and stem cells in HFDP cells in response to porphyra-334 treatment.
