# Supplementary material for: Transcriptome Profiling of Human Follicle Dermal Papilla Cells in response to Porphyra-334 Treatment by RNA-Seq
Source: Evid Based Complement Alternat Med. 2021 Jan 13;2021:6637513. doi: 10.1155/2021/6637513 (PMC7817261; doi:10.1155/2021/6637513)
Supplement: Supplementary Materials — Table S1: enriched GO terms in 27 genes in HFDP cells upregulated by porphyra-334 treatment. Table S2: expression of selected genes involved in hair follicle structure, epidermal structure, and stem cells in HFDP cells in response to porphyra-334 treatment. [file 6637513.f1.zip › 6637513.f1/Supplementary table1.pdf]

Table S1. Enriched GO terms in 27 genes in HFDP cells upregulated by porphyra-334 treatment.
In order to identify enriched GO terms, 27 up-regulated genes were subjected to overrpresentation analysis against GO database using WebGestalt program.
We identified enriched GO terms from two categories, biological process and cellular component.

Functional category	Gene Set	Description	P-value	FDR	Gene	Size	Expect	Ratio
Biological process	GO:0010273	detoxification of copper ion	2.6256E-07	0.00091698	MT1E, MT1X, MT2A	14	0.013441	223.21
Biological process	GO:1990169	stress response to copper ion	2.6256E-07	0.00091698	MT1E, MT1X, MT2A	14	0.013441	223.21
Biological process	GO:0061687	detoxification of inorganic compound	3.2801E-07	0.00091698	MT1E, MT1X, MT2A	15	0.014401	208.32
Biological process	GO:0097501	stress response to metal ion	4.0347E-07	0.00091698	MT1E, MT1X, MT2A	16	0.015361	195.3
Biological process	GO:0071294	cellular response to zinc ion	1.1056E-06	0.0020103	MT1E, MT1X, MT2A	22	0.021121	142.04
Biological process	GO:0071280	cellular response to copper ion	1.8623E-06	0.0028217	MT1E, MT1X, MT2A	26	0.024961	120.19
Biological process	GO:0006882	cellular zinc ion homeostasis	4.6633E-06	0.0055856	MT1E, MT1X, MT2A	35	0.033601	89.282
Biological process	GO:0071276	cellular response to cadmium ion	5.0843E-06	0.0055856	MT1E, MT1X, MT2A	36	0.034561	86.802
Biological process	GO:0055069	zinc ion homeostasis	5.5297E-06	0.0055856	MT1E, MT1X, MT2A	37	0.035521	84.456
Biological process	GO:0046688	response to copper ion	8.1461E-06	0.006786	MT1E, MT1X, MT2A	42	0.040322	74.402
Biological process	GO:0007059	chromosome segregation	0.000008211	0.006786	PTTG1, NEK2, NCAPG, MAD2L1, BIF	312	0.29953	16.693
Biological process	GO:0000070	mitotic sister chromatid segregation	0.000012007	0.009096	MAD2L1, NCAPG, NEK2, PTTG1	155	0.14881	26.881
Biological process	GO:0010043	response to zinc ion	0.000017477	0.011751	MT1E, MT1X, MT2A	54	0.051842	57.868
Biological process	GO:0036018	cellular response to erythropoietin	0.000018096	0.011751	MT1X, MT2A	7	0.0067203	297.61
Biological process	GO:0000819	sister chromatid segregation	0.000024099	0.013701	MAD2L1, NCAPG, NEK2, PTTG1	185	0.17761	22.522
Biological process	GO:0036017	response to erythropoietin	0.000024114	0.013701	MT1X, MT2A	8	0.0076803	260.41
Biological process	GO:0046686	response to cadmium ion	0.000027834	0.014885	MT1E, MT1X, MT2A	63	0.060482	49.601
Biological process	GO:0098813	nuclear chromosome segregation	0.000088197	0.044544	MAD2L1, NCAPG, NEK2, PTTG1	258	0.24769	16.149
Cellular component	GO:0000779	condensed chromosome, centromeric region	8.8197E-07	0.0010363	BIRC5, MAD2L1, NCAPG, NEK2	117	0.079791	50.131
Cellular component	GO:0000775	chromosome, centromeric region	6.6172E-06	0.0038876	BIRC5, MAD2L1, NCAPG, NEK2	194	0.1323	30.234
Cellular component	GO:0000793	condensed chromosome	0.000011477	0.0044953	BIRC5, MAD2L1, NCAPG, NEK2	223	0.15208	26.302
Cellular component	GO:0000777	condensed chromosome kinetochore	0.00004245	0.01247	BIRC5, MAD2L1, NEK2	104	0.070925	42.298
Cellular component	GO:0098687	chromosomal region	0.000054021	0.012695	BIRC5, MAD2L1, NCAPG, NEK2	331	0.22573	17.72
Cellular component	GO:0000776	kinetochore	0.000088361	0.017304	BIRC5, MAD2L1, NEK2	133	0.090702	33.075
Cellular component	GO:0044427	chromosomal part	0.00018838	0.031621	BIRC5, E2F1, MAD2L1, NCAPG, NEK	886	0.60423	8.275
