# Supplementary material for: Transcriptome Profiling of Human Follicle Dermal Papilla Cells in response to Porphyra-334 Treatment by RNA-Seq
Source: Evid Based Complement Alternat Med. 2021 Jan 13;2021:6637513. doi: 10.1155/2021/6637513 (PMC7817261; doi:10.1155/2021/6637513)
Supplement: Supplementary Materials — Table S1: enriched GO terms in 27 genes in HFDP cells upregulated by porphyra-334 treatment. Table S2: expression of selected genes involved in hair follicle structure, epidermal structure, and stem cells in HFDP cells in response to porphyra-334 treatment. [file 6637513.f1.zip › 6637513.f1/Supplementary table2.pdf]

Table S2. Expression of selected genes involved in hair follicle structure, epidermal structure, and stem cells in HFDP cells in response to porphyra-334 treatment.

Hair follicle structure-associated genes

Gene ID	Gene function	baseMean	log2FoldChange	P-value
CDH3	P-Cadherin 3	1.972539	2.352451	0.249678
GATA6	GATA binding protein 6	2012.237	-0.5197	0.043474
KRT16	Keratin 16	4.06513	-1.58742	0.208571
KRT17	Keratin 17	27.31534	0.13691	0.788896
KRT33B	Keratin 33B	4.877546	0.636122	0.589267
KRT34	Keratin 34	21.82566	1.244568	0.035363
KRT79	Keratin 79	12.33744	4.20037	3.91E-04
KRT86	Keratin 86	4.725019	-0.03207	0.979703
PMEL	Premelanosome protein	5.261059	-0.60939	0.536367
TCHH	Trichohyalin	2.239498	2.543768	0.200154

Epidermal structure-associated genes.

Gene ID	Gene function	baseMean	log2FoldChange	P-value
DSC1	Desmocollin 1	11.62712	-1.03332	0.15108
DSG1	Desmoglein 1	2.823108	5.023933	0.030943
DSG2	Desmoglein 2	128.4983983	-0.607325504	0.354204545
DSG3	Desmoglein 3	32.05270241	1.294435542	0.535545419
DSP	Desmoplakin	2500.301	-1.07603	0.005958
FLG	Filaggrin	23.19803	-1.16759	0.101662
KRT1	Keratin 1	1.911986	0.957471	0.675418
KRT10	Keratin 10	529.3117	0.131666	0.619732
KRT14	Keratin 14	189.1779	-0.03752	0.941173
PLEC	Plectin	866.1326	-0.79877	0.020143
TP63	Tumor protein p63	1.599167	-0.37925	0.839795

Stem Cell-Associated Genes

Gene ID	Gene function	baseMean	log2FoldChange	pvalue
CD200	CD200 Molecule	22.10806418	-0.185929848	0.726151448
CD34	CD34 Molecule	8.918307741	1.08233539	0.347419679
ITGA6	Integrin subunit alpha 6	171.5685697	0.007712293	0.977171358
ITGB1	Integrin subunit beta 1	73242.08306	0.022729305	0.905573
KRT15	Keratin 15	16.71504707	1.889454979	0.014987849
KRT19	Keratin 19	498.2867514	0.070218029	0.857094957
NFATC1	Nuclear factor of activated T cells	46.06128931	0.002997251	0.993888262
SOX9	SRY-box transcription factor 9	657.0633614	1.232453382	0.001630333
TCF3	Transcription factor 3	54.373916	0.405991893	0.273993229
TCF4	Transcription factor 4	28.65397441	-0.410120476	0.352579822
